# Effectiveness and safety of remimazolam combined with alfentanil in hysteroscopic examination: A prospective, randomized, single-blind trial

**DOI:** 10.1097/MD.0000000000037627

**Published:** 2024-04-12

**Authors:** Bei Huang, Nan-Ping Li, Gang-Kai Tan, Na Liang

**Affiliations:** a The Affiliated Nanhua Hospital, Hengyang Medical School, University of South China, Hengyang, Hunan, China; b The Affiliated Nanhua Hospital, Department of Anesthesiology, Hengyang Medical School, University of Suth China, Hengyang, Hunan, China.

**Keywords:** Alfentanil, anesthesia, ED50, hysteroscopy, propofol, remimazolam

## Abstract

**Background:**

Remimazolam is a novel, ultrashort-acting benzodiazepine. This study aimed to compare the efficacy and safety of remimazolam and propofol for hysteroscopic examination, to determine the optimal dose of remimazolam combined with alfentanil for painless hysteroscopy, and to calculate its median effective dose (ED50).

**Methods:**

Step 1: A total of 208 patients undergoing hysteroscopic examination were prospectively included in this study. Patients were randomized into 4 groups: 0.2 mg/kg remimazolam (group A), 0.25 mg/kg remimazolam besylate (group B), 0.3 mg/kg remimazolam (group C), and 2 mg/kg propofol (group D), with 52 patients in each group. One minute after losing consciousness, patients received an intravenous injection of alfentanil at 5 µg/kg, followed by a continuous infusion of alfentanil at 0.5 µg/kg/min. If patients showed frowning, movement, or MOAA/S > 1, sedatives were added: 0.05 mg/kg/dose of remimazolam for groups A, B, and C, and 0.5 mg/kg/dose of propofol for group D. Step 2: Dixon’s up-and-down method was used to calculate the ED50 of remimazolam combined with alfentanil during hysteroscopic examination.

**Main results:**

The sedation success rates of the remimazolam groups were 88.46%, 94.23%, and 98.08%, respectively, compared to 96.15% in the propofol group, with no significant difference (*P* = .175). MAP in groups A and B was higher than in group D (*P* < .05), and significantly higher in group C than in group D (*P* = .0016). SpO2 values in groups A, B, and C were higher than in group D at T2 to T3 (*P* < .001). HR in groups A, B, and C was significantly higher than in group D (*P* < .001). The ED50 of remimazolam combined with alfentanil in hysteroscopy was 0.244 mg/kg, 95%CI (0.195–0.22) and ED95 was 0.282 mg/kg, 95%CI (0.261–1.619).

**Conclusion:**

In hysteroscopy, the sedative effect of remimazolam is like that of propofol, with 0.25 mg/kg remimazolam showing better safety and efficacy, and less impact on the respiratory and circulatory systems. Additionally, under the influence of alfentanil, the ED50 of remimazolam in hysteroscopy is 0.244 mg/kg, with no severe adverse reactions observed.

## 1. Introduction

Gynecological disorders, such as abnormal uterine bleeding, endometrial polyps,^[1]^ endometrial hyperplasia, and cancer, are common and increasingly prevalent. Hysteroscopy considered a minimally invasive procedure for the diagnosis and treatment of various gynecological diseases, has become the preferred method for examining uterine bleeding and intrauterine pathology.^[2–5]^ The process of hysteroscopy, including dilation, distention, and curettage, can cause some pain to the patients. Therefore, a safe and painless anesthetic state is essential for the smooth conduct of hysteroscopic surgery.

Propofol can alleviate pain caused by surgical stimulation such as uterine expansion and swelling, but it may cause adverse reactions like respiratory depression, hypotension, bradycardia, and injection pain. Moreover, Nolan et al^[6]^ found that propofol has a dose-dependent suppressive effect on respiratory and circulatory functions, which, though clinically manageable, can lead to severe adverse events if overlooked or not promptly identified. Therefore, balancing the anesthetic effects and adverse reactions of propofol is a pressing issue. In hysteroscopic surgeries, propofol is used in combination with opioids, dexmedetomidine, and ketamine. Studies show that low-dose sufentanil and propofol are the most common outpatient intravenous anesthesia regimens, despite severe respiratory depression^[7]^; ketamine combination significantly reduces respiratory and circulatory suppression but often causes schizophrenia-like symptoms^[8,9]^; combining with dexmedetomidine improves sedation and analgesic effects with fewer respiratory side effects but can lead to prolonged hypotension or bradycardia.^[10]^ Clearly, these therapeutic methods have their drawbacks.

Remimazolam, a new benzodiazepine drug, is one of the ultra-short-acting sedatives.^[11]^ It works by enhancing the activity of GABAa receptors with γ subunits. It acts on central GABAa receptors, opens channels, increases chloride ion inflow, and causes hyperpolarization of the neuronal membrane, thus inhibiting neuronal activity.^[12]^ Due to its rapid onset, water solubility, short half-life (about 0.75 hours), and having a specific antagonist (flumazenil), it is becoming a common drug for sedation in short surgeries. However, remimazolam alone does not meet the clinical needs of hysteroscopic surgery and needs to be used in combination with analgesics. Clinical studies by Zhang et al^[13]^ showed that remimazolam combined with low-dose sufentanil for hysteroscopic surgery still exhibits many adverse reactions, such as respiratory depression. Alfentanil, a synthetic fentanyl derivative acting on μ-opioid receptors, is a short-acting potent analgesic. A prospective randomized study showed that remimazolam combined with alfentanil has the advantages of good analgesic effect, quick onset, quick consciousness recovery, and short waking time in colonoscopic surgery.^[14]^ Consequently, the proposed integration of remimazolam and alfentanil presents a promising avenue for exploration in the domain of hysteroscopic surgery.

This study aims to explore the combined use of remimazolam and alfentanil in painless hysteroscopic examination, determine the optimal dose, and calculate the effective median dose of remazolam under the influence of alfentanil. We conducted a randomized controlled study to compare the safety and efficacy of different doses of remimazolam combined with alfentanil in painless hysteroscopic examination. We hypothesized that the sedation success rate of remimazolam is not inferior to that of propofol.

## 2. Methods

### 2.1. Sample size

**Figure 1.**
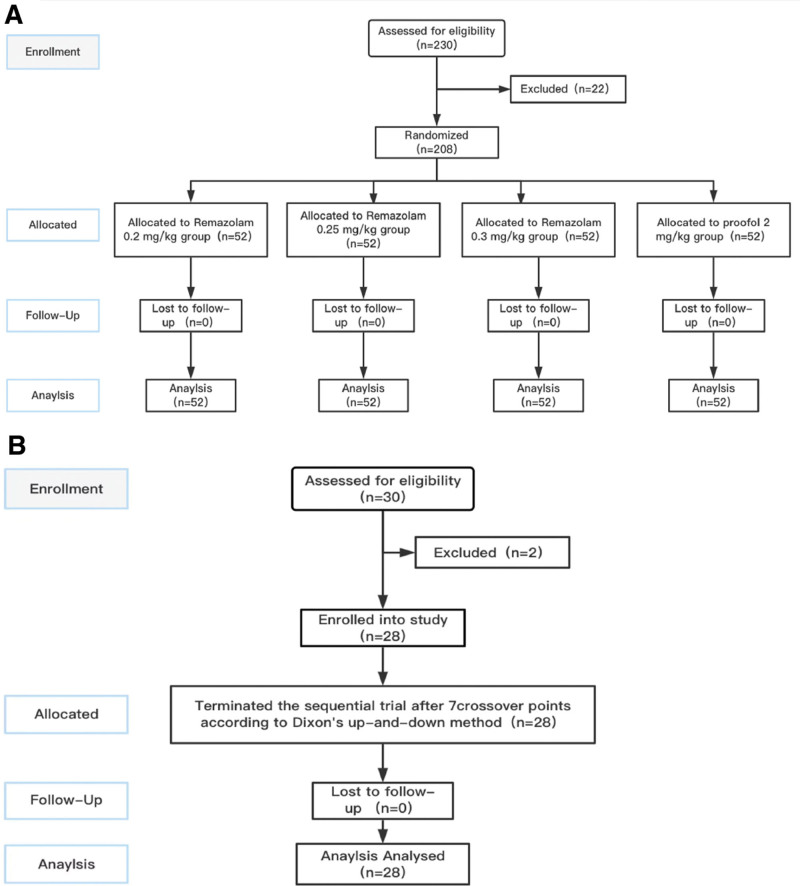
Flow diagram of the study (A). Flow diagram of the ED50 (B).

This study was conducted from May to November 2023. Based on previous studies and preexperiments, we assumed a 100% sedation success rate for both remimazolam and propofol in hysteroscopic examinations.^[15]^ In our trial, the combination of propofol and alfentanil served as a positive control. For the primary outcome (sedation success rate), the predefined noninferiority margin was set at 0.6 with a power of 90% and a 1-sided *α* of 2.5%. The ratio of patients in the remimazolam groups (0.2, 0.25, 0.3 mg/kg) to the propofol group was 3:1, resulting in a required sample size of 41 patients per group (Supplemental diagram). Assuming a dropout rate of 20%, 52 patients per group were needed to be recruited. Through screening, a total of 208 patients were ultimately included as the sample size for this study (Fig. 1A).

The sample size was considered adequate once 7 pairs of reversal of sequence had been achieved when deciding the median effective dose using an up-and-down sequential method (Fig. 1B).

The study protocol was subject to approval by Ethics Committee of Nanhua Hospital, University of South China (No. 2023-KY-48). The formulation of this study scheme was in accordance with the requirements of the Declaration of Helsinki of the World Medical Association. All participants signed the informed consent. The study was registered at the Chinese Clinical Trial Registry (No. ChiCTR2300071424).

### 2.2. Randomization and blinding

Due to the distinct colors of propofol and remimazolam, making them easily distinguishable, this study adopted a single-blind method. Randomization was conducted by researchers not involved in anesthesia management or perioperative follow-up to prevent selection bias. A randomized number list was used to divide patients into 4 groups. Random numbers were sealed in opaque envelopes, and patients were included based on these envelopes. One researcher, unaware of the study protocol and trained in assessment methods prior to the study, conducted preoperative assessments and postoperative follow-ups. An anesthesiologist handled anesthesia management and intraoperative data collection. A statistician analyzed the final data. All researchers, except the anesthesiologist, were blinded to the group assignments.

A total of 230 female patients were randomly selected for hysteroscopic examination at Nanhua Hospital affiliated with Nanhua University, with 208 completing the study. The study flowchart (Fig. 1A) shows patients divided into 4 groups, each with 52 patients. Experimental groups (remimazolam groups): groups A, B, and C were induced with 0.2, 0.25, and 0.3 mg/kg doses of remimazolam, respectively^[16]^; Control group: group D was induced with 2 mg/kg propofol.^[17]^

Another random selection of 30 female patients underwent hysteroscopic examination at the same hospital, with 28 completing the study. The study flowchart is depicted in Figure 1B.

### 2.3. Inclusion and exclusion criteria

#### 2.3.1. Inclusion criteria

Female patients aged 25 to 65 (most of the patients coming to the hospital were over 25 and over 25 and more decision-making); body mass index (BMI) between 18 and 30 kg/m²; preoperative consciousness, intravenous general anesthesia, and spontaneous breathing; preoperative American Society of Anesthesiologists (ASA) classification I to II; no severe dysfunction of heart, lung, brain, or other vital organs; normal liver and kidney function; clear understanding of the study and voluntary participation with signed informed consent.

#### 2.3.2. Exclusion criteria

Severe dysfunction of heart, lung, liver, kidney, or severe cardiovascular diseases; heart rate (HR) < 55 bpm or high-degree atrioventricular block requiring a pacemaker; sustained sinus tachycardia or severe arrhythmias; hypotension, shock, or hypertension stage 2 or above; known allergies to anesthetics used in the study or severe allergy history; long-term use of nonsteroidal antiinflammatory drugs, narcotic analgesics, or sedatives; mental health disorders (schizophrenia, mania, confusion), long-term psychiatric medication, and cognitive impairments; no serious adverse events occurred during the study, enrolled or unexpected events; patients with severe respiratory lesions (obstructive sleep apnea syndrome, acute respiratory tract infection, acute onset of chronic obstructive pulmonary disease, uncontrolled asthma, etc) or myasthenia gravis; participated in any clinical trial as a subject in the last 3 months; patients who are not considered appropriate by the investigator to participate in the trial.

### 2.4. Anesthesia procedure

No premedication was administered before anesthesia induction. Upon entering the operating room, patients were positioned in the lithotomy position and given mask oxygen inhalation at 5 L/min for 5 minutes before induction. Monitoring included mean arterial pressure (MAP), HR, oxygen saturation (SpO2), bispectral index (BIS), and Nociception Index (NOX) until patients woke from anesthesia and left the operating room.

The anesthesiologist recorded necessary experimental data, such as age, height, and weight. In the experimental groups: groups A, B, and C were administered remimazolam at doses of 0.2, 0.25, and 0.3 mg/kg, respectively; Control group: group D received 2.0 mg/kg propofol intravenously. All groups completed the drug administration within 1 minute. One minute later, the patients lost consciousness and received an intravenous injection of alfentanil at 5 µg/kg, followed by a continuous infusion of 0.5 µg/kg/min.^[18]^ If patients frown, move, or MOAA/ S > 1,^[19]^ adding sedative medication: remimazolam at 0.05 mg/kg/time in groups A, B, and C, and propofol at 0.5 mg/kg/time in group D. Sedation success refers to the addition of sedative medication no more than 5 times in each group. The sedation in each group was added no more than 5 times. If frowning, exercise or MOAA/ S > 1 occurred after 5 times, the sedation failed. The remedial measure is to continue adding sedation until the end of the procedure.

In case of hypoxia or respiratory depression (SpO2 < 90% for more than 10 seconds), the oxygen flow was increased, and jaw thrust was performed. If SpO2 did not improve, the hysteroscopic examination was paused, and mild chest compression was applied. If the situation did not improve, assisted ventilation with a jaw thrust mask or intubation was performed. Vasoactive drugs like norepinephrine, ephedrine, atropine, and urapidil were used to maintain hemodynamic stability.

The study used the Dixon sequential method. The initial dose of remimazolam was 0.25 mg/kg (from the above experiment), with a gradient of 0.01 mg/kg according to Dixon and Massey’s sequential allocation rules. If conditions for cervical dilation and hysteroscope placement were poor, or if there was a positive response (defined as any physical movement or frowning within 2 minutes after entering the uterus), the dose was increased by 0.01 mg/kg. If there were no positive responses, the dose for the next patient was decreased by 0.01 mg/kg. The experiment concluded when each group achieved 7 crossovers.

All patients were treated by a single attending anesthesiologist, and all surgeries were performed by the same doctor. Recorded data included respiratory depression, initial dose of remimazolam, total dose administered, surgical time, and recovery time (from the last administration to awakening).

### 2.5. Observation index

#### 2.5.1. Primary observation indicators

Observing the sedation success rate of groups A, B, C, and D, and the medications failing to achieve successful sedation.

#### 2.5.2. Secondary observation indicators

Recording changes in HR, MAP, and SpO2 at different time points: time entering the operating room (T1), time of sedative induction (T2), 1 minute after administering the sedative (T3), time entering hysteroscopy (T4), 2 minutes after entering the uterus (T5), during curettage (T6), end of surgery (T7), recovery time (T8), and time leaving the operating room (T9). Recording MOAA/S, BIS, and NOX scores, adverse events, surgical duration, alfentanil dosage, and total dosage of sedatives for T1-T9.

The MOAA/S scale is a 6-point scale, and it is described as 5: responds readily to name spoken in normal tone; 4: lethargic response to name spoken in normal tone; 3: responds only after the name is called loudly and/or repeatedly; 2: responds only after mild prodding or shaking; 1: responds only after painful trapezius squeeze; 0: no response after painful trapezius squeeze. After the MOAA/S score was < 1, the surgeon was allowed to begin placement of the vaginal speculum, which signaled the start of the operation.

The BIS was used to assess patients’ level of sedation.^[20]^ Previous studies found that the depth of sedation determined by BIS and MOAA/S methods was similar. Recent studies have emphasized the clinical benefits of using electroencephalography for monitoring, significantly correlating with sedation scale scores, and offering more convenience as it does not require patient stimulation.^[21,22]^ However, the BIS index was initially developed for propofol, and its accuracy in assessing benzodiazepines like remimazolam is lower.^[23]^ Therefore, BIS serves as a reference indicator for remimazolam sedation level. The NOX was used to assess the level of analgesia, focusing on pain nociceptive stimuli and brain function. NOX detection provided firsthand clinical data for the rational use of opioids and minimizing nociceptive stimuli. Thus, NOX was used in this study to monitor pain during hysteroscopy and observe the analgesic effects of alfentanil.

### 2.6. Statistical analysis

In this study, SPSS software version 27.0 (IBM SPSS Statistics Inc., Chicago, IL, USA) was used for statistical analysis. Quantitative data are presented as mean ± standard deviation. Qualitative data are presented as the chi-square test. Normally distributed continuous variables at different time points were compared with paired *t* tests. The ED50 and ED95 of remimazolam, along with their 95% confidence intervals (CIs), were calculated using the sequential method. The ED50 of remimazolam was calculated as the average of midpoints of ineffective–effective crossovers. The ED95 (95%CI) was estimated by using probit regression. For all analyses, *P* < .05 was considered to indicate statistically significant differences. Graphs were created using GraphPad Prism version 5.0 (GraphPad Software Inc., San Diego, CA, USA).

## 3. Results

### 3.1. The safety and efficacy of remimazolam is not inferior to propofol

#### 3.1.1. Demographic data

In all, 230 patients were enrolled and screened. Among them, 208 patients were divided randomly into 4 groups and included in the final analysis (Fig. 1). The demographic characteristics of the patients are given in Table 1. The characteristics were similar in all groups.

**Table 1:** Data are presented as the mean ± standard deviation or the number of patients.

Characteristics of patients	A group (*n* = 52)	B group (*n* = 52)	C group (*n* = 52)	D group (*n* = 52)	*P* value
Age (yr)	50.02 ± 10.82	47.02 ± 10.61	47.98 ± 11.78	47.73 ± 11.29	.290
Height (cm)	158.1 ± 4.14	158.37 ± 5.16	157.06 ± 4.36	157.69 ± 4.63	.396
Weight (kg)	58.27 ± 9.22	58.6 ± 9.7	55.54 ± 6.88	57.73 ± 8.71	.219
BMI (kg/m^2^)	23.32 ± 3.53	23.33 ± 3.47	22.52 ± 2.77	23.21 ± 3.29	.332
ASA status					
I/II	33/18	34/19	38/14	35/17	.757
Allergic history	1 (1.92)	2 (3.85)	2 (3.85)	1 (1.92)	.876
Total dose of remimazolam (mg)	21.01 ± 4.67	23.02 ± 4.36	22.12 ± 3.26	NA	.050
Total dose of propofol (mg)	NA	NA	NA	66.32 ± 79.7	NA
Total alfentanil dose(mg)	0.66 ± 0.16	0.72 ± 0.16	0.7 ± 0.15	0.7 ± 0.16	.106
Awakening duration(min)	5.37 ± 1.47**	6.88 ± 1.62**	8.06 ± 1.56	8.71 ± 1.88	.000**
Operation time(min)	16.17 ± 4.13	15.94 ± 4.92	15.4 ± 4.46	15.99 ± 4.65	.717
Repair	6 (11.54)	3 (5.77)	1 (1.92)	2 (3.85)	.175
The sedation success rate	88.46%	94.23%	98.08%	96.15%	.175

** *P* < 0.01.

ASA = American Society of Anesthesiologists, BMI = body mass index, HR = heart rate, MAP = mean arterial pressure.

Group A received remazolam 0.2 mg/kg. Group B received remazolam 0.25 mg/kg. Group C received remazolam 0.3 mg/kg. Group D received propofol 2 mg/kg.

**Figure 2.**
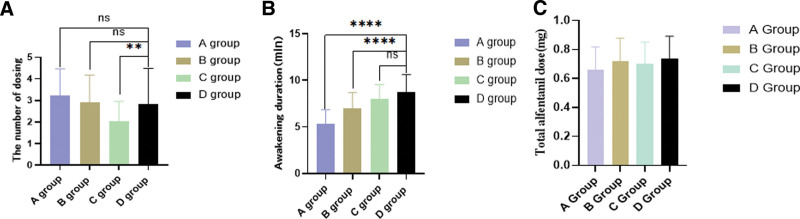
Intraoperative dosing times (A), awakening duration (B), and total dose of alfentanil (C). (*P < .0 5, **P < .01, ****P < .001, ****P < .0001).

As shown in Fig. 2A, compared with group D, the number of intraoperative group C sedative increases was significantly decreased (*P* < .01), while there was no significant difference in the number of sedations added in groups A and B, (*P* < .01), indicating that the high dose of remimazolam could make the operation smooth and have better sedative effect. According to Table 1, there was no significant difference in the duration of surgery among the 4 groups (*P* = .717). The recovery time in group A (5.37 ± 1.47 minutes) and in group B (6.88 ± 1.62 minutes) was significantly shorter than that in group D (8.71 ± 1.88 minutes) (*P* < .001), while that in group C was not significantly different from group D (Fig. 2B). This suggests that the recovery time with remimazolam is faster than with propofol, but as the dose of remimazolam increases, the recovery time also lengthens, eventually becoming like that of propofol.

Table 1 shows that the total dose of remimazolam (21.01 ± 4.67 mg) was compared with group B (23.02 ± 4.36 mg) and group C (22.12 ± 3.26 mg), not statistically significant (*P* = .05) (Fig. 2C). The total alfentanil dose was not significantly different between the 4 groups (*P* = .106).

#### 3.1.2. Main results

**Figure 3.**
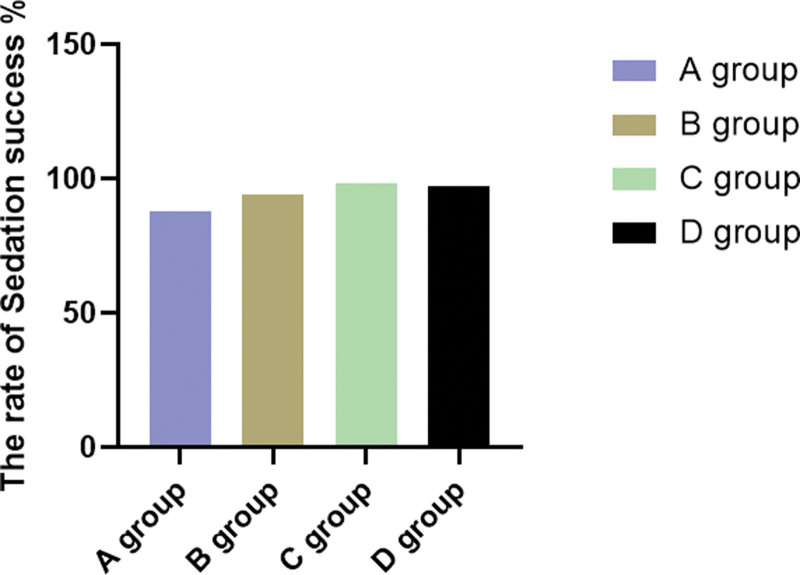
Sedation success rate.

As shown in Table 1, compared with sedation success in group D (96.15%), group A was 88.46%, 94.23% in group B and 98.08% in group C, and 4 groups were not statistically significant (*P* > .05) (Fig. 3). This indicates that in hysteroscopic surgery, there is no significant difference in the sedation success rate between the remimazolam and propofol groups, and the difference is not greater than the noninferiority margin. Therefore, the sedative effect of remimazolam is noninferior to propofol.

#### 3.1.3. Secondary outcomes

**Figure 4.**
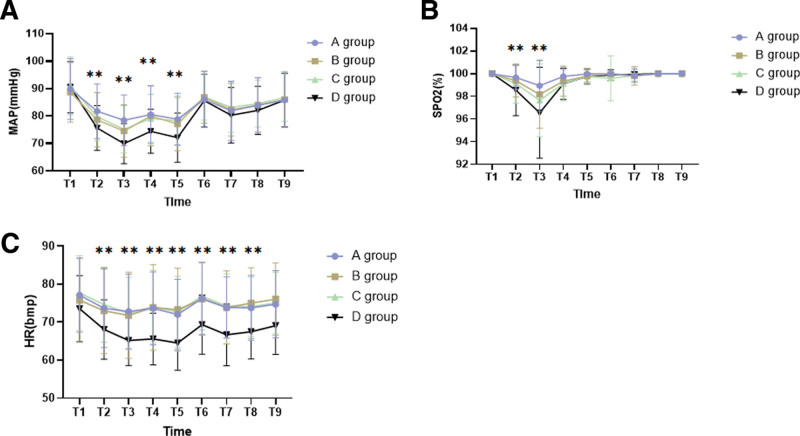
Changes in vital signs over time. MAP = mean arterial pressure (A), SpO2 = oxygen saturation (B), HR = heart rate (C). (*P < .05, **P < .01, ***P < .001, ****P < .0001).

As shown in Fig. 4A, MAP in groups A and B was higher than that in D (*P* < .05), and MAP in group C was significantly higher than that in group D (*P* = .0016). This illustrates fewer hemodynamic fluctuations in the remimazolam group compared to the propofol group. At T1, MAP, HR, and SpO2 were insignificant between the remimazolam and propofol groups (*P* > .05). However, during T2 to T5, the MAP values in groups A, B, and C were significantly higher than those in group D, with all differences being statistically significant (*P* < .001). At T2 to T3, SpO2 values were higher in groups A, B, and C compared to group D, and were statistically significant (*P* < .001) (Fig. 4B). Therefore, the probability of respiratory depression in the remimazolam group was lower than that in the propofol group. Moreover, the HR of groups A, B, and C was significantly higher than that of group D (*P* < .001) (Fig. 4C). The smaller fluctuations in MAP, HR, and SpO2 in the remimazolam groups, compared to the propofol group, suggest that remimazolam effectively reduces the impact on respiratory and cardiovascular systems.

**Figure 5.**
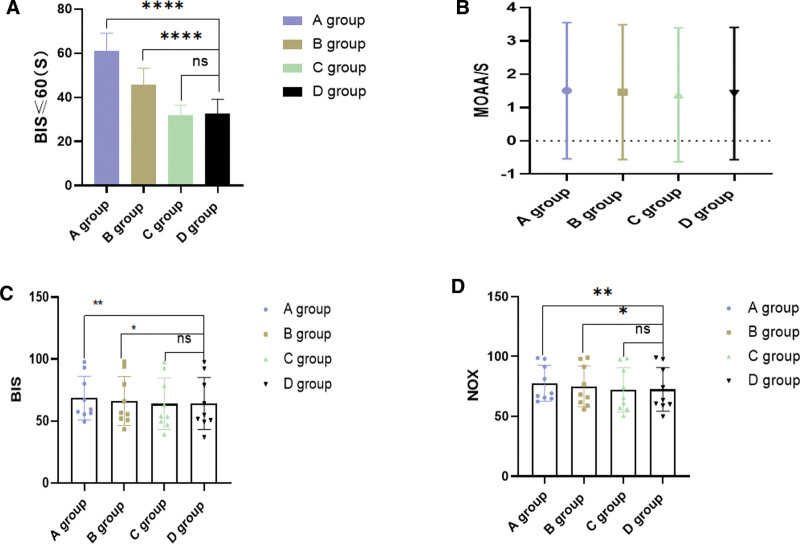
BIS values reached 1 min after induction with Remimazolam or propofol (A), MOAA/ S sedation score (B), BIS = the bispectral index (C), and NOX = Nociception Index (D). (*P < .05, **P < .01, ***P < .001, ****P < .0001).

One minute after induction with remimazolam used alone, as the dosage of remimazolam increased, the time to reach a sedated state (BIS ≤ 60) was significantly reduced, as shown in Figure 5A (*P* < .001). In this study, there was no significant difference in the rate of sedation success among patients, and none required any additional medication or withdrawal from the study due to insufficient depth of anesthesia. As illustrated in Figure 5B, there were no significant differences in MOAA/S scores between the 4 groups (*P* > .05), indicating that the depth of anesthesia was adequately effective as per MOAA/S scoring. As shown in Figure 5C, compared to group D, groups A and B had higher BIS values, while there was no significant difference between groups C and D (*P* > .05). As indicated in Figure 5D, the NOX values in groups A and B were significantly higher than in group D, with a statistically significant difference (*P* < .05). However, there was no significant difference in NOX values between groups C and D. In summary, with a constant dose of alfentanil, the depth of sedation and analgesia increased with higher doses of remimazolam, suggesting a dose-dependent synergistic effect of remimazolam and alfentanil.

#### 3.1.4. Safety analysis

No fatal adverse events occurred in any group during the procedure. As shown in Table 2, there was no significant difference in the incidence of dizziness in the 4 groups (*P* > .05); as hysteroscopic surgery progressed, sedation and analgesia gradually became insufficient, leading to minor physical movements in patients. However, these did not affect the surgery or lead to withdrawal from the study. The incidence of body movement in the remimazolam groups was significantly lower than in the propofol group (*P* = .027). The incidence of respiratory depression in group A (9.62%) and that of respiratory depression in group B (11.54%) were significantly lower than those in group C (22.92%) and group D (34.48%) (*P* = .001). The incidence of respiratory depression was significantly lower in the remimazolam group than in the propofol group. Injection pain mostly occurred in group D (25%). Injection pain was the most common adverse event in the propofol group, but it did not occur in the remimazolam group (*P* < .001). The incidence of hiccups in group A (15.38%), group B (21.15%), and group C (38.46%) were significantly higher than group D (1.92%) (*P* < .001). Thus, hiccup was a common adverse event seen in the remimazolam group. Follow-up of the patient showed no statistically significant POVN at 1 and 24 hours after surgery (*P* = .063) (Table 2).

**Table 2:** Data are presented as the mean ± standard deviation or the number of patients.

Untoward effect	A group (*n* = 52)	B group (*n* = 52)	C group (*n* = 52)	D group (*n* = 52)	*P* value
Body movement	31 (59.62)	26 (50.00)	16 (30.77)	26 (50.00)	0.027*
Dizzy	0 (0.00)	3 (5.77)	8 (15.38)	12 (23.08)	0.001**
Hiccup	8 (15.38)	11 (21.15)	20 (38.46)	1 (1.92)	0.000**
Injection pain	0 (0.00)	0 (0.00)	0 (0.00)	13 (25.00)	0.000**
Respiratory depression	5 (9.62)	6 (11.54)	11 (22.92)	20 (34.48)	0.001**
Postoperative follow-up					
PONV	0 (0.00)	1 (1.92)	2 (3.82)	5 (9.62)	0.063

* *P* < 0.05,

** *P* < 0.01.

PONV = postoperative nausea and vomiting.

Group A received remazolam 0.2 mg/kg. Group B received remazolam 0.25 mg/kg. Group C received remazolam 0.3 mg/kg. Group D received propofol 2 mg/kg.

The above data indicate that compared to the propofol group, the remimazolam group showed better sedation effects, faster recovery, lower incidence of respiratory depression, more stable hemodynamics, and a lower incidence of adverse reactions. However, as the dose of remimazolam increased, while sedation effects enhanced, the rate of adverse reactions also continued to rise. In conclusion, 0.25 mg/kg of remimazolam combined with alfentanil is more suitable for hysteroscopic surgery.

### 3.2. The ED50 of remimazolam combined with alfentanil in hysteroscopy

The above experiments concluded that remimazolam and alfentanil have a synergistic effect on sedation and analgesia, and their combined use can reduce opioid-related reactions. Therefore, this study aimed to test the influence of alfentanil on the induction dose ED50 of remimazolam in hysteroscopic examinations and to determine the optimal dose, which to our knowledge, has not been explored in previous studies.

*Demographic data.* A total of 30 patients were recruited, with 28 completing the experiment. The flowchart for ED50 is depicted in Fig. 1B. The demographic characteristics of the patients in this group are shown in Table 3.

**Table 3:** Data are presented as the mean ± standard deviation or the number of patients.

Characteristics of patients	R group (*n* = 28)
Age (yr)	42 ± 8.028
Height (cm)	157.241 ± 5.827
Weight (kg)	58.143 ± 8.343
BMI (kg/m^2^)	23.504 ± 2.863
MAP (mm Hg)	75.595 ± 5.376
SBP (mm Hg)	100.857 ± 7.189
DBP (mm Hg)	62.964 ± 5.693
HR (bpm)	66.821 ± 7.898
SpO2 (%)	98.786 ± 2.363

ASA = American Society of Anesthesiologists, BMI = body mass index, DBP = diastolic blood pressure, HR = heart rate, MAP = mean arterial pressure, SBP = systolic blood pressure, SpO2 = oxygen saturation.

**Figure 6.**
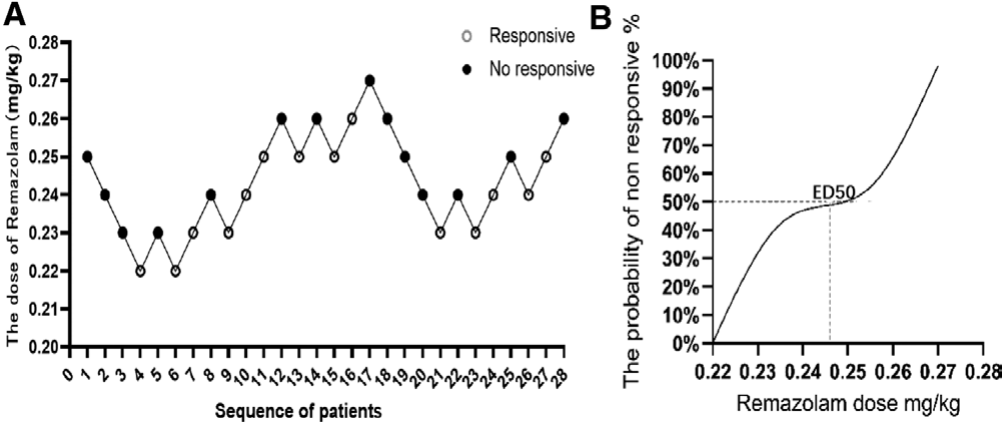
The sequential trial of remimazolam (A); the dose–response curve of remimazolam (B).

Figure 6A presents the ED50 and ED95 (95% confidence interval) of remimazolam in this group, based on the Dixon-Massey up-and-down sequential allocation method and logistic regression. The ED50 of remimazolam combined with alfentanil in hysteroscopic examination was calculated to be 0.244 mg/kg, with a 95%CI of 0.195–0.22 mg/kg, and ED95 was 0.282 mg/kg, with a 95%CI of 0.261–1.619 mg/kg. Figure 6B shows the dose–response analysis of remimazolam combined with alfentanil in hysteroscopic examination. Thus, the median effective dose of remimazolam for induction was 0.244 mg/kg.

## 4. Discussion

Remimazolam is metabolized by hepatic esterases and rapidly eliminated by carboxylesterases into an inactive carboxylic acid metabolite, CNS7054, with 1/400th of the sedative effect.^[24]^ This metabolic pathway and pharmacokinetics of its metabolites contribute to remimazolam’s rapid onset and systemic clearance, independent of age and disease state.^[23,25]^ Worthington first reported the reversal of remimazolam-induced sedation with flumazenil in human studies, and the availability of this antagonist is an advantage of remimazolam over other intravenous anesthetics like propofol.^[26,27]^ Remimazolam offers sedation, hypnosis, and anxiolysis with rapid onset and clearance, painless injection, minimal respiratory and cardiovascular suppression, and low liver and kidney function dependence. Its clinical application is expanding, as demonstrated in phase III clinical studies by Chen et al for colonoscopy and gastroscopy, where propofol served as a noninferiority control.^[28]^ Remimazolam showed sedation success not inferior to propofol, with lower incidences of hypotension and respiratory depression, indicating its safety and efficacy in gastrointestinal endoscopy. Pastis et al found remimazolam sedation to be safe and effective during bronchoscopy in a multicenter prospective study.^[29]^ Zhang et al^[15]^ observed that both remimazolam and propofol had a 100% success rate, but remimazolam had faster recoveries, less hemodynamic fluctuation, and low SpO2 compared to propofol, indicating its advantages in hysteroscopic surgery.

Remimazolam was developed to exploit in a benzodiazepine the esterase pharmacology successfully deployed in the remifentanil. However, alfentanil instead of remifentanil was invoked during analgesia because of the higher incidence of hyperalgesia and muscle stiffness in remifentanil.^[30,31]^ Our study results show that propofol or remimazolam combined with alfentanil can quickly achieve the sedation depth required for hysteroscopic examination. However, the remimazolam group had fewer adverse reactions than the propofol group postsedation initiation, indicating higher safety when sedation success rates are similar.

A study suggested that remifentanil enhances remimazolam’s sedative effect and remimazolam possibly remifentanil’s analgesic effect, showing a synergistic action with opioids.^[32]^ Our study indicates that with increasing remimazolam doses, lower BIS and NOX values, fewer body movements, longer awakening times, and better sedative-analgesic effects are observed, suggesting a synergistic effect of alfentanil deepening remimazolam’s sedation.

Compared with the propofol group, hiccups often occurred in the remimazolam group. In one continuous infusion study, the incidence of hiccups was low in the remimazolam group^[27]^; another study found that multiple hiccups were observed after infusion induction.^[23,25]^ Therefore, the incidence of hiccups may also be related to the rate or method of remimazolam administration during induction. Another potential factor contributing to the occurrence of hiccups could be the classification of remimazolam as a benzodiazepine.^[33,34]^ Existing research indicates a correlation between benzodiazepines, as well as corticosteroids, with increased incidence of hiccups. These compounds are postulated to be among the most frequent pharmaceutical agents implicated in triggering hiccups.^[35]^ Some studies have shown that midazolam also causes hiccups that can be reversed with flumazenil.^[36]^ However, the mechanism by which benzodiazepines cause hiccups remains unclear. Hiccups might be related to GABA neurotransmitters, as benzodiazepines stimulate various effects via GABA. Hiccups during surgery are a safety concern but are self-limiting within minutes and do not impact the procedure.

In clinical anesthesia, early determination of ED50 and ED95 is important to determine the administered dose.^[37,38]^ The Dixon sequential method is one of the common methods used to calculate the effective dose/ concentration of drugs. Its advantages include minimal sample size, use of concentrated test doses, high efficiency, and ease of implementation.^[39]^ In our study, Dixon’s sequential method was used, starting with a preset dose of 0.25 mg/kg and a dose step of 0.01 mg/kg. The ED50 of remimazolam and alfentanil was 0.244 (0.195–0.22) mg/kg, and the ED95 was 0.282 (0.261–1.619) mg/kg for hysteroscopic examination suppression. This study showed only 1 patient experienced low SpO2 with no other adverse reactions, possibly explaining the minimal respiratory impact of low-dose remimazolam.

This study has limitations: our study population was entirely female, so the response of 0.25 mg/kg remimazolam in males requires further study; the study only recruited ASA I and II patients, so the safety and efficacy of remimazolam in higher-risk groups need further exploration; ED95 is derived from ED50, so more research is needed to obtain more accurate data; this study was single-centered and focused solely on hysteroscopic examinations, which limits its generalizability. Therefore, the results of this study need to be further confirmed through large-scale, multicenter, randomized, controlled trials.

## 5. Conclusion

During hysteroscopic examinations, remimazolam has been proven to be a safer and more effective anesthetic choice. Overall, 0.25 mg/kg of remimazolam combined with alfentanil offers the best safety and efficacy for hysteroscopic examinations, having a lesser impact on respiratory and circulatory systems compared to propofol. Additionally, the ED50 of remimazolam combined with alfentanil under hysteroscopic conditions is 0.244 (0.195–0.22) mg/kg, and the ED95 is 0.282 (0.261–1.619) mg/kg. This study, being single-center, suggests the need for multicenter research to draw more relevant conclusions.

## Acknowledgments

We would like to thank all participants who contributed their time to the study.

## Author contributions

**Conceptualization:** Bei Huang.

**Data curation:** Bei Huang, Nan-Ping Li, Gang-Kai Tan, Na Liang.

**Formal analysis:** Bei Huang, Gang-Kai Tan.

**Funding acquisition:** Na Liang.

**Investigation:** Nan-Ping Li, Gang-Kai Tan.

**Resources:** Bei Huang, Nan-Ping Li.

**Supervision:** Na Liang.

**Writing—original draft:** Bei Huang.

**Writing—review & editing:** Bei Huang.

## References

[1] Boretto M Maenhoudt N Luo X. Patient-derived organoids from endometrial disease capture clinical heterogeneity and are amenable to drug screening. Nat Cell Biol. 2019;21:1041–51. 31371824 10.1038/s41556-019-0360-z

[2] Vitale SG Bruni S Chiofalo B. Updates in office hysteroscopy: a practical decalogue to perform a correct procedure. Updates Surg. 2020;72:967–76. 32008214 10.1007/s13304-020-00713-w

[3] Vilà Famada A Cos Plans R Costa Canals L. Outcomes of surgical hysteroscopy: 25 years of observational study. J Obstet Gynaecol. 2022;42:1365–9. 34913810 10.1080/01443615.2021.1971176

[4] Donnez J Nisolle M. Hysteroscopic surgery. Curr Opin Obstet Gynecol. 1992;4:439–46. 1623154

[5] Kolhe S. Management of abnormal uterine bleeding—focus on ambulatory hysteroscopy. Int J Womens Health. 2018;10:127–36. 29606892 10.2147/IJWH.S98579 PMC5868607

[6] Nolan PJ Delgadillo JA Youssef JM. Dexmedetomidine provides fewer respiratory events compared with propofol and fentanyl during third molar surgery: a randomized clinical trial. J Oral Maxillofac Surg. 2020;78:1704–16. 32554067 10.1016/j.joms.2020.05.015

[7] Xiao X Xiao N Zeng F. Gastroscopy sedation: clinical trial comparing propofol and sufentanil with or without remimazolam. Minerva Anestesiol. 2022;88:223–9. 35072431 10.23736/S0375-9393.21.15917-6

[8] Zhan Y Liang S Yang Z. Efficacy and safety of subanesthetic doses of esketamine combined with propofol in painless gastrointestinal endoscopy: a prospective, double-blind, randomized controlled trial. BMC Gastroenterol. 2022;22:391. 35987996 10.1186/s12876-022-02467-8 PMC9392938

[9] Yang H Zhao Q Chen HY. The median effective concentration of propofol with different doses of esketamine during gastrointestinal endoscopy in elderly patients: a randomized controlled trial. Br J Clin Pharmacol. 2022;88:1279–87. 34496448 10.1111/bcp.15072

[10] Tekeli AE Oğuz AK Tunçdemir YE. Comparison of dexmedetomidine-propofol and ketamine-propofol administration during sedation-guided upper gastrointestinal system endoscopy. Medicine (Baltim). 2020;99:e23317. 10.1097/MD.0000000000023317 PMC7717792 33285707

[11] Kim KM. Remimazolam: pharmacological characteristics and clinical applications in anesthesiology. Anesth Pain Med (Seoul). 2022;17:1–11. 35139608 10.17085/apm.21115 PMC8841266

[12] Rogers WK Mcdowell TS. Remimazolam, a short-acting GABA(A) receptor agonist for intravenous sedation and/or anesthesia in day-case surgical and non-surgical procedures. IDrugs. 2010;13:929–37. 21154153

[13] Zhang S Wang J Ran R. Efficacy and safety of remimazolam tosylate in hysteroscopy: a randomized, single-blind, parallel controlled trial. J Clin Pharm Ther. 2022;47:55–60. 34655087 10.1111/jcpt.13525

[14] Xin Y Chu T Wang J. Sedative effect of remimazolam combined with alfentanil in colonoscopic polypectomy: a prospective, randomized, controlled clinical trial. BMC Anesthesiol. 2022;22:262. 35974309 10.1186/s12871-022-01805-3 PMC9380378

[15] Zhang X Li S Liu J. Correction to: Efficacy and safety of remimazolam besylate versus propofol during hysteroscopy: single-centre randomized controlled trial. BMC Anesthesiol. 2021;21:173. 34144683 10.1186/s12871-021-01390-x PMC8212503

[16] Pambianco DJ Borkett KM Riff DS. A phase IIb study comparing the safety and efficacy of remimazolam and midazolam in patients undergoing colonoscopy. Gastrointest Endosc. 2016;83:984–92. 26363333 10.1016/j.gie.2015.08.062

[17] Doi M Morita K Takeda J. Efficacy and safety of remimazolam versus propofol for general anesthesia: a multicenter, single-blind, randomized, parallel-group, phase IIb/III trial. J Anesth. 2020;34:543–53. 32417976 10.1007/s00540-020-02788-6

[18] Xu K Huang Y Tan X. Use of remimazolam combined with alfentanil for plastic surgery anesthesia cases: a clinical trial. Ann Plast Surg. 2023;90(5S Suppl 2):S221–4. 36752399 10.1097/SAP.0000000000003377

[19] Chernik DA Gillings D Laine H. Validity and reliability of the observer’s assessment of alertness/sedation scale: study with intravenous midazolam. J Clin Psychopharmacol. 1990;10:244–51. 2286697

[20] Toklu S Iyilikci L Gonen C. Comparison of etomidate-remifentanil and propofol-remifentanil sedation in patients scheduled for colonoscopy. Eur J Anaesthesiol. 2009;26:370–6. 19300267 10.1097/EJA.0b013e328318c666

[21] Yamamoto T Kurabe M Kamiya Y. A mechanism of re-sedation caused by remimazolam. J Anesth. 2021;35:467–8. 33822281 10.1007/s00540-021-02930-y

[22] Liu X Ding B Shi F. The efficacy and safety of remimazolam tosilate versus etomidate-propofol in elderly outpatients undergoing colonoscopy: a prospective, randomized, single-blind, non-inferiority trial. Drug Des Devel Ther 2021;15:4675–85. 10.2147/DDDT.S339535 PMC8606755 34819721

[23] Schüttler J Eisenried A Lerch M. Pharmacokinetics and Pharmacodynamics of Remimazolam (CNS 7056) after continuous infusion in healthy male volunteers: part I. Pharmacokinetics and Clinical Pharmacodynamics. Anesthesiology. 2020;132:636–51. 31972655 10.1097/ALN.0000000000003103

[24] Kilpatrick GJ Mcintyre MS Cox RF. CNS 7056: a novel ultra-short-acting Benzodiazepine. Anesthesiology. 2007;107:60–6. 17585216 10.1097/01.anes.0000267503.85085.c0

[25] Sheng XY Liang Y Yang XY. Safety, pharmacokinetic and pharmacodynamic properties of single ascending dose and continuous infusion of remimazolam besylate in healthy Chinese volunteers. Eur J Clin Pharmacol. 2020;76:383–91. 31873765 10.1007/s00228-019-02800-3

[26] Worthington MT Antonik LJ Goldwater DR. A phase Ib, dose-finding study of multiple doses of remimazolam (CNS 7056) in volunteers undergoing colonoscopy. Anesth Analg. 2013;117:1093–100. 24108261 10.1213/ANE.0b013e3182a705ae

[27] Chen X Sang N Song K. Psychomotor recovery following remimazolam-induced sedation and the effectiveness of flumazenil as an antidote. Clin Ther. 2020;42:614–24. 32178858 10.1016/j.clinthera.2020.02.006

[28] Chen SH Yuan TM Zhang J. Remimazolam tosilate in upper gastrointestinal endoscopy: a multicenter, randomized, non-inferiority, phase III trial. J Gastroenterol Hepatol. 2021;36:474–81. 32677707 10.1111/jgh.15188

[29] Pastis NJ Yarmus LB Schippers F. PAION Investigators. Safety and efficacy of remimazolam compared with placebo and midazolam for moderate sedation during bronchoscopy. Chest. 2019;155:137–46. 30292760 10.1016/j.chest.2018.09.015

[30] Kops MS Pesic M Petersen KU. Impact of concurrent remifentanil on the sedative effects of remimazolam, midazolam and propofol in cynomolgus monkeys. Eur J Pharmacol. 2021;890:173639. 33065095 10.1016/j.ejphar.2020.173639

[31] Sneyd JR Rigby-Jones AE. Remimazolam for anaesthesia or sedation. Curr Opin Anaesthesiol. 2020;33:506–11. 32530890 10.1097/ACO.0000000000000877

[32] Bevans T Deering-Rice C Stockmann C. Inhaled remimazolam potentiates inhaled remifentanil in rodents. Anesth Analg. 2017;124:1484–90. 28333705 10.1213/ANE.0000000000002022

[33] Micallef J Tardieu S Pradel V. Benzodiazepine and hiccup: three case reports. Therapie. 2005;60:57–60. 15929474 10.2515/therapie:2005007

[34] Tang S Lu J Xu C. Feasibility and safety of remazolam versus propofol when inserting laryngeal masks without muscle relaxants during hysteroscopy. Drug Des Devel Ther. 2023;17:1313–22. 10.2147/DDDT.S408584 PMC10162397 37152102

[35] Bor S Mandiracioglu A Kitapcioglu G. Gastroesophageal reflux disease in a low-income region in Turkey. Am J Gastroenterol. 2005;100:759–65. 15784016 10.1111/j.1572-0241.2005.41065.x

[36] Arroyo-Cózar M Grau Delgado J Gabaldón Conejos T. Hiccups induced by midazolam during sedation in flexible bronchoscopy. Arch Bronconeumol. 2012;48:103. 22265321 10.1016/j.arbres.2011.11.005

[37] Zhang J Kong L Ni J. ED50 and ED95 of propofol combined with different doses of intravenous lidocaine for first-trimester uterine aspiration: a prospective dose-finding study using up-and-down sequential allocation method. Drug Des Devel Ther. 2022;16:3343–52. 10.2147/DDDT.S382412 PMC9527702 36199630

[38] Yu J Xiang B Song Y. ED50 of propofol in combination with low-dose sufentanil for intravenous anaesthesia in hysteroscopy. Basic Clin Pharmacol Toxicol. 2019;125:460–5. 31231918 10.1111/bcpt.13280

[39] Tan M Zhang C Zeng W. Determining the effective dose of esketamine for mitigating pain during propofol injection by Dixon’s up-and-down method: a double-blind, prospective clinical study of drug dose response. BMC Anesthesiol. 2022;22:368. 36457068 10.1186/s12871-022-01914-z PMC9714076

